# α_2_-adrenoreceptor modulated FAK pathway induced by dexmedetomidine attenuates pulmonary microvascular hyper-permeability following kidney injury

**DOI:** 10.18632/oncotarget.10809

**Published:** 2016-07-24

**Authors:** Qian Chen, Bin Yi, Jianbo Ma, Jiaoling Ning, Lingzhi Wu, Daqing Ma, Kaizhi Lu, Jianteng Gu

**Affiliations:** ^1^ Department of Anesthesiology, Southwest Hospital, Third Military Medical University, Chongqing, China; ^2^ Anaesthetics, Pain Medicine and Intensive Care, Department of Surgery and Cancer, Faculty of Medicine, Imperial College London, Chelsea & Westminster Campus, London, United Kingdom

**Keywords:** dexmedetomidine, α2-adrenoreceptor, FAK, endothelial barrier, lung injury, Pathology Section

## Abstract

Renal ischemia-reperfusion (rI/R) could cause remote acute lung injury (ALI) and combination of these two organ injuries can remarkably increase the mortality. This study aims to determine whether dexmedetomidine, an α_2_-adrenoreceptor agonist sedative, can ameliorate pulmonary microvascular hyper-permeability following rI/R injury and explore the underlying mechanisms. In *vivo*, C57BL/6J mice received dexmedetomidine (25μg/kg, i.p.) in the absence or presence of α_2_-adrenergic antagonist atipamezole (250μg/kg, i.p.) or focal adhesion kinase (FAK) inhibitor (30mg/kg, i.p.) before bilateral renal pedicle clamping for 45 minutes followed by 24 hours reperfusion. The lung histopathological changes and the permeability of pulmonary microvascular were assessed respectively. In *vitro*, the cultured C57BL/6J mice pulmonary microvascular endothelial cells (PMVECs) were treated with serum from mice with rI/R with or without dexmedetomidine and atipamezole. Trans-endothelial permeability and phospho-tyrosine^397^FAK, F-actin, VE-cadherin and ZO-1 in monolayer PMVECs were measured respectively in the presence or absence of rI/R serum, dexmedetomidine and FAK inhibitor. In *vivo*, dexmedetomidine remarkably attenuated lung injury and pulmonary microvascular hyper-permeability caused by rI/R injury, which was abolished by atipamezole or FAK inhibitor co-administration. *In vitro*, the permeability of PMVECs monolayer following exposure to serum from rI/R mice was increased significantly, and decreased by dexmedetomidine. Dexmedetomidine increased phospho-tyrosine^397^FAK in a time- and dose-dependent manner, which was correlated with the changes in trans-endothelial permeability. Our data indicated that dexmedetomidine is able to ameliorate remote pulmonary microvascular hyper-permeability induced by rI/R, at least in part, *via* FAK modulation.

## INTRODUCTION

Acute kidney injury (AKI) is a frequent complication amongst the critical ill patients especially following major cardiovascular surgery [1-3]. Previous accumulated studies have demonstrated that AKI induced by ischemia/reperfusion (I/R) leads to dysfunction of the extra-renal organs including lung, heart, brain, small intestine and liver [4-7]. Due to its large microcapillary network, the lung is highly susceptible to damage caused by circulating pro-inflammatory or pro-apoptosis mediators from kidney inflicted with I/R [8]. The mortality of combined AKI and acute lung injury (ALI) even reaches up to 80% in critical care settings [9].

Pulmonary microvascular endothelial cells (PMVECs) are important to maintain the homeostasis of pulmonary and cardiovascular systems. The various circulating mediators produced following AKI might directly activate the pulmonary signaling pathways, including pro-inflammatory, pro-apoptotic and vascular-reaction responses in the lung. Lung edema from pulmonary microvascular endothelial barrier disruption is one of the hallmark features of ALI. Hence, maintenance and repair of PMVECs barrier might become an effective treatment strategy for AKI-induced ALI.

Dexmedetomidine is a highly selective α_2_-adrenoreceptor agonist with sedative, analgesic, sympatholytic, hemodynamic stabilizing and diuretic properties, and it has been widely used in the operating rooms and intensive care units (ICU) [10]. Recent studies [11-15], either *in vivo* or *in vitro*, demonstrated that dexmedetomidine exerted potential protective effects against lung, kidney, brain, intestine and heart injuries induced by I/R injury or other insults. Our previous study even demonstrated that pre- or post-treatment with dexmedetomidine remarkably attenuated renal ischemia/reperfusion (rI/R)-associated pulmonary edema through its potential anti-inflammatory property [8]. Additionally, dexmedetomidine has been reported to activate the neural focal adhesion kinase (FAK) [16], a cytoplasmic protein tyrosine kinase that plays a central role in initiating and integrating various signaling pathways to affect F-actin and several crucial proteins that tightly seal adjacent endothelial cells, e.g. zonula occludens-1 (ZO-1) and vascular-endothelial cadherin (VE-cadherin). Such proteins are important to maintain the vascular barrier integrity and restrict paracellular permeability [17, 18]. The present study investigates the hypothesis that dexmedetomidine decreases microvascular hyper-permeability *via* endothelial FAK signaling to preserve PMVECs morphology and function, and ultimately protects the lungs, when distant AKI occurs.

## RESULTS

### Dexmedetomidine prevents rI/R induced lung injury *in vivo*

**Figure 1:**
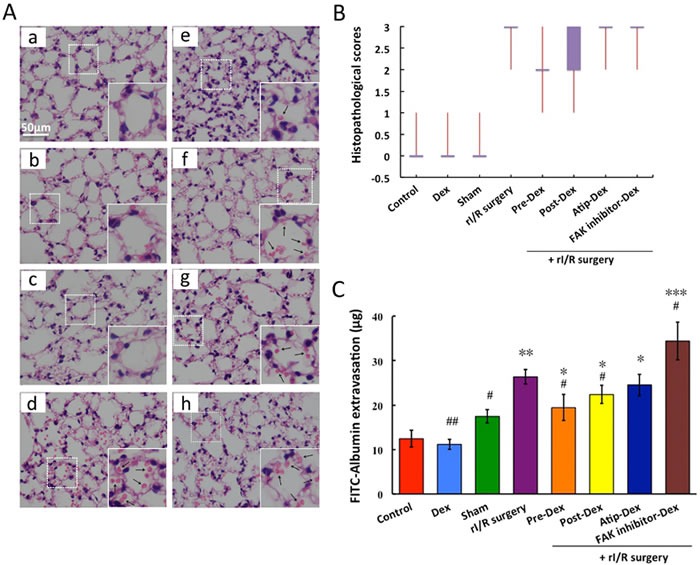
Dexmedetomidine prevents rI/R induced lung injury and pulmonary microvascular hyper-permeability *in vivo* C57BL/6J mice were pre-treated or post-treated with dexmedetomidine (Dex) alone or in combination with a_2_-adrenoceptor antagonist atipamezole (Atip) or FAK inhibitor 14 (inhibit FAK phosphorylation)followed by clamping of the bilateral renal pedicle for 45 minutes and reperfusion 24 hours. **A.** Example micrographs of lung tissue section under difference conditions (a: Control; b: Dex; c: Sham; d:rI/R surgery; e: Pre-Dex + rI/R surgery; f: Post-Dex + rI/R surgery; g: Atip-Dex + rI/R surgery; h: FAK inhibitor-Dex + rI/R surgery; Arrows show the erythrocytes); **B.** Lung injurious scores presented with Box-Whiskers plot; **C.** The FITC-Albumin extravasation into lung tissue. Data are mean ± SD (*n* = 5-6);*p < 0.05, **p < 0.01, ***p < 0.001*vs*.Control, ^#^*p* < 0.05, ^##^*p* < 0.05*vs*.rI/R surgery.

Whilst the alveolar structures were integral, without any erythrocytes exudation in the Control, dexmedetomidine-treated or Sham group (Figure 1Aa, b and c), pulmonary structure was almost completely damaged in the rI/R groups (Figure 1Ad), with remarkable cell infiltration and significant erythrocytes leak into alveolar space. Intraperitoneal injection of dexmedetomidine at 25 μg/kg before or after rI/R significantly reduced the lung injury and erythrocytes leakage (Figure 1Ae and 1Af). Co-treatment with the α_2_-adrenoceptor antagonist atipamezole or FAK inhibitor 14 abolished the effect of dexmedetomidine (Figure 1Ag and 1Ah). All these were corroborated with the mean pattern changes in the various groups (Figure 1B).

Regarding pulmonary microvascular permeability, compared to the control group (12.43 ± 0.86 μg), the FITC-Albumin extravasation in the rI/R group was significantly increased (26.35 ± 1.66 μg) (*p* < 0.01). Pre-and post-treatment with dexmedetomidine resulted in 26% (19.43 ± 2.97 μg, *p* < 0.05) and 15% (22.36 ± 2.03 μg, *p* < 0.05) respective reductions of the leak of FITC-Albumin when compared that in the rI/R group. Its effect was abolished by α_2_-adrenoceptor antagonist atipamezole (24.54 ± 2.42 μg; *vs*. control, *p* < 0.05) or FAK inhibitor 14 (34.4 ± 4.25 μg; *vs*. control, *p* < 0.05), suggesting dexmedetomidine confers protection to lung *via* α_2_-adrenoceptor or FAK pathway.

### Dexmedetomidine reduced the hyper-permeability of PMVECs monolayer treated with rI/R serum

**Figure 2:**
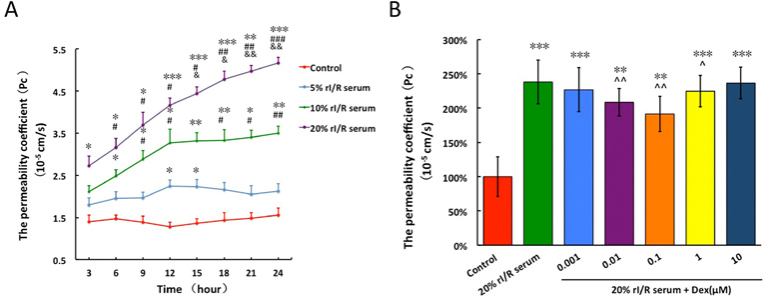
The effects of rI/R serum on the permeability of PMVECs monolayer and the modulated effects of dexmedetomidine on the permeability The PMVECs monolayer was treated with mice normal serum or different concentrations of (5%, 10%, 20%) rI/R serum and their permeability coefficient was assessed; **B.** 20% rI/R serum was used to test the effects of different concentrations (0.001μM~10μM) of dexmedetomidine on the permeability of PMVECs monolayer. Data are expressed as mean ± SD (*n* = 4-6); *p < 0.05, **p < 0.01,***p < 0.001*vs*. Control; ^#^*p* < 0.05,^##^*p* < 0.01,^###^*p* < 0.001 *vs*. 5% rI/R serum; ^&^*p* < 0.05,^&&^*p* < 0.01 *vs*. 10% rI/R serum. ^^^*p* < 0.05,^^^^*p* < 0.01*vs*.20% rI/R serum.

The permeability coefficient (Pc) of PMVECs monolayer treated with 20% rI/R serum continued to increase remarkably from 3 hours (Pc = 2.7 ± 0.2 × 10^−5^ cm/s *vs*. control, *p* < 0.05) to 24 hours (Pc = 4.97 ± 0.15 × 10^−5^ cm/s, *vs*. control, *p* < 0.001). The Pc of 10% rI/R serum treated group increased to 3.27 ± 0.31 × 10^−5^ cm/s (*vs*. control, *p* < 0.05; *vs*. 5% rI/R serum, *p* < 0.05) at 12 hours and reached a plateau. Though 5% rI/R serum also increased Pc when compared to that of normal serum treated group, the increase was not significant (Figure 2A). Therefore, 20% rI/R serum was used in the following experiments.

Compared to the control group (Pc = 1.88 ± 0.07 × 10^−5^ cm/s), the Pc of rI/R serum treated group was increased by 2.47 fold (Pc = 4.84 ± 0.13 × 10^−5^ cm/s, *p* < 0.001). Interestingly, dexmedetomidine treatment to 20% rI/R serum induced endothelial barrier hyper-permeability produced dose-dependent, bidirectional changes. The permeability of dexmedetomidine-treated groups fell at concentrations ranged 0.001 to 0.1 μM, however permeability rose when at higher concentration between 0.1 and 10 μM. The PMVECs monolayer hyper-permeability was attenuated by 18.9% and 17.7% from 0.01 μM (Pc = 3.89 ± 0.17 × 10^−5^ cm/s;*vs*. control, *p* < 0.01; *vs*. 20% rI/R serum, *p* < 0.01) and 0.1μM (Pc = 3.98 ± 0.18 × 10^−5^ cm/s; *vs*. control, *p* < 0.01; *vs*. 20% rI/R serum, *p* < 0.01) dexmedetomidine, respectively (Figure 2B).

### FAK activity in PMVECs in the presence of dexmedetomidine

**Figure 3:**
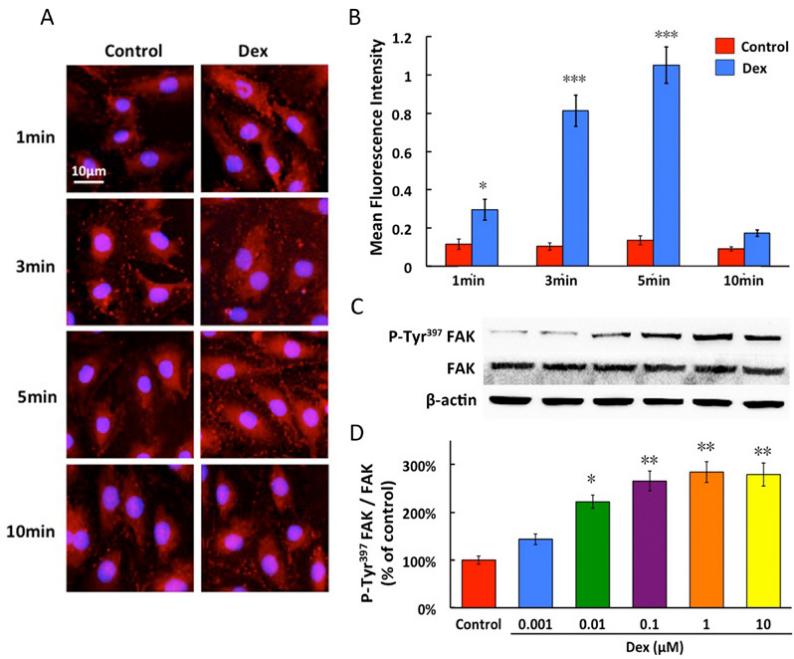
Time-dependent (A and B) and concentration-dependent(C and D) effects of dexmedetomidine on FAK phosphorylation of PMVECs Dexmedetomidine acts in a time-dependent fashion from 1 to 10 minutes and concentration-dependent manner from 0.001 to 10μM, promoted the protein expression of P-Tyr^397^FAK in PMVECs. Data are expressed as the percentage of control (mean ± SD, *n* = 4-6).*p < 0.05, **p < 0.01, ***p < 0.001*vs*. Control.

0.1 μM dexmedetomidine led to a linear increase in FAK phosphorylation between 1 and 5 minutes. The amount of P-Tyr^397^FAK declined to the baseline at 10 minutes (Figure 3A and 3B). In PMVECs, P-Tyr^397^FAK molecules were distributed across the cells in a punctuated, dot-like pattern, with a preferable localization at the cell surface.

In western blot study, dexmedetomidine resulted in a concentration-dependent increase in expression of P-Tyr^397^FAK in PMVECs and its phosphorylated level nearly peaked, when normalized to total FAK expression, with dexmedetomidine at 0.1 μM. In contrast, dexmedetomidine did not significantly increase the expression of total FAK (Figure 3C and 3D).

### FAK activation reduced the hyper-permeability induced by rI/R serum

**Figure 4:**
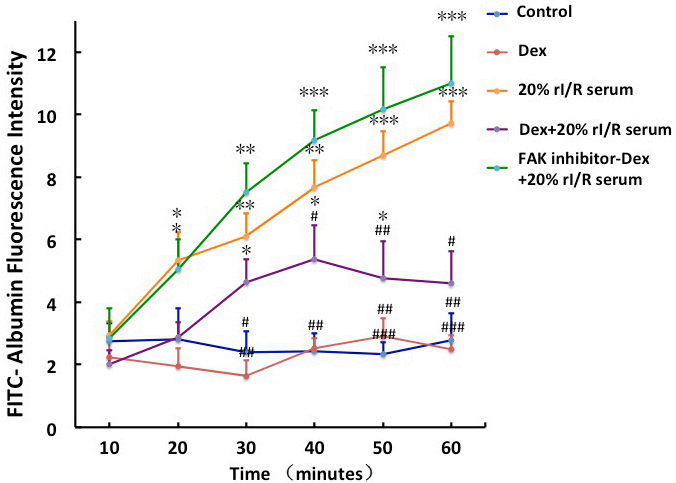
The effect of FAK on the rI/R serum induced hyper-permeability of pulmonary endothelial monolayers The endothelial monolayers in transwell chambers were continuously incubated with 10μM FAK inhibitor 14 for 3hours before 0.1μM dexmedetomidine treatment for 20 minuets, followed by 20% rI/R serum stimuli for 60 minutes. Fluorescence (FITC-Albumin, 100mg/mL) was measured at every 10minutes. Data are expressed as mean ± SD (*n* = 5); *p < 0.05, **p < 0.01,***p < 0.001*vs*. Control;^#^*p* < 0.05,^##^*p* < 0.01,^###^*p* < 0.001*vs*.20% rI/R serum.

The FITC-Albumin fluorescence intensity increased gradually from 10 minutes and peaked at 60 minutes with 3.48-fold increase with 20% rI/R serum compared to that in the control (*p* < 0.001). Dexmedetomidine pretreatment significantly decreased the permeability of the PMVECs monolayer exposed to rI/R serum. Pretreatment with FAK inhibitor 14 for 3 hours reversed the permeability reduction of monolayer by dexmedetomidine, to imply that the mechanism of dexmedetomidine-mediated decline in PMVECs monolayer hyper-permeability is likely to involve FAK phosphorylation (Figure 4).

### Dexmedetomidine maintains cell cytoskeleton and cell junction *via* FAK activity

**Figure 5:**
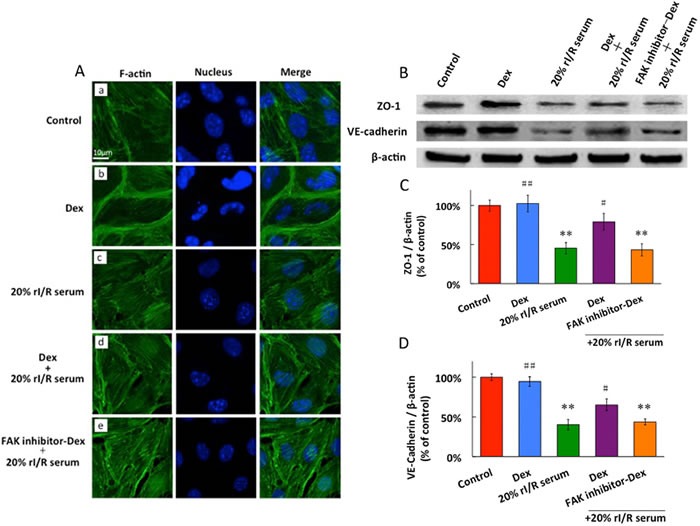
The effects of rI/Rserum on F-actin cytoskeletal assembly **A.,** and the ZO-1 and VE-cadherin expressions in PMVECs (B, C, D). PMVECs incubated with 10μM FAK inhibitor for 3 hours before 0.1μM dexmedetomidine treatment for 20 minutes, followed by 20% rI/R serum stimulation for 60 minutes and F-actin was detected by Immunofluorescence. FITC-phalloidin fluorescence of F-actin cytoskeletal assembly with DAPI staining for the nucleus in PMVECs: **A.** No visible increase in stress fiber formation in the normal cell; **B.** dexmedetomidine significantly enhanced cortical cytoskeleton staining; **C.** 20% rI/R serum induced dissolution of cortical cytoskeleton and formation of prominent stress fiber and intercellular gaps; **D.** Pretreatment with dexmedetomidine prevented rI/R serum induced actin stress fiber formation, and diminished the paracellular gaps ; **E.** FAK inhibitor reversed the effect of dexmedetomidine. **B.** ZO-1 and **C.** VE-cadherin were detected by western blot. Data are expressed as the percentage of control (mean ± SD, *n* = 4);**p < 0.01*vs*.Control;^#^*p* < 0.05, ^##^*p* < 0.01*vs*.20% rI/R serum.

The endothelial cell cytoskeleton is a critical determinant of vascular integrity and barrier regulation and is known to be influenced by FAK [19]. We examined the effect of dexmedetomidine and rI/R serum on spatial localization and polymerization of F-actin by immunofluorescence microscopy. The control endothelial cells exhibited a fine central reticular actin pattern and there was no visible increase in stress fiber formation (Figure 5Aa). Dexmedetomidine treatment was associated with rapid and dramatic enhancement of polymerized F-actin staining that was spatially confined to the cortical cytoskeletal ring, which correlated with the enhancement of endothelial barrier function (Figure 5Ab). Stimulation with 20% rI/R serum for 1 hour induced dissolution of the cortical cytoskeleton, and the F-actin was prominently organized into axially oriented stress fibers in many of the observed cells, to result in the appearance of small paracellular gaps (Figure 5Ac). Dexmedetomidine pretreatment for 20 minutes before 1 hour rI/R serum cleared the F-actin in PMVECs from the center of the cells to be localized at the cell periphery, to diminish the paracellular gaps (Figure 5Ad). The similar pattern of actin as of Figure 5Ac appeared when PMVECs were treated with FAK inhibitor for 3 hours, followed by dexmedetomidine for 20 minutes and 1 hour 20% rI/R serum (Figure 5Ae).

The PMVECs constitutively express ZO-1, VE-cadherin under control conditions and dexmedetomidine has no influence on ZO-1 and VE-cadherin baseline expressions. 20% rI/R serum led to significant down regulation that approximated to 55% in ZO-1 expression and 59% in VE-cadherin expression, compared to those in the control group (*p* < 0.01, respectively). Pretreatment with dexmedetomidine for 60 minutes significantly increased ZO-1 and VE-cadherin expressions by 34% and 26%, respectively (*vs*. 20% rI/R serum, *p* < 0.01, respectively). Pretreatment with FAK inhibitor 14 for 3 hours reversed dexmedetomidine-associated upregulation in ZO-1 and VE-cadherin proteins expressions (*vs*. control, *p* < 0.01, respectively) (Figure 5B, 5C and 5D).

## DISCUSSION

Our work showed that dexmedetomidine ameliorated pulmonary microvascular hyper-permeability following rI/R and its effect was abolished by α_2-_adrenoreceptor antagonist atipamezole or FAK inhibitor, indicating that dexmedetomidine acted in an α_2-_adrenoreceptor and/or FAK-dependent manner. Our *in vitro* experiments also demonstrated that dexmedetomidine improved PMVECs barrier function through activation of α_2-_adrenoreceptor/FAK pathway, which corresponded to reduction in rI/R serum-induced actin stress fiber formation and increase in cell junction complex proteins (ZO-1 or VE-cadherin) expression.

Pre- and post-treatment with dexmedetomidine at 25 μg/kg both attenuated FITC-Albumin leaking from lung vasculature following rI/R serum stimulation, and pre-treatment seemed to be more effective. As previous demonstrated, dexmedetomidine possesses potential anti-inflammatory and anti-apoptosis properties and hence provided organ protection [20, 21]. The systematic inflammatory responses triggered by inflammatory factors, such as tumor necrosis factor-α (TNF-α), interleukin-1β (IL-1β), IL-6 and macrophage inflammatory protein (MIP-1), when released into the circulation due to rI/R, can directly damage lung [22]. The anti-inflammatory effect of dexmedetomidine was proved to be an important mechanism of pulmonary protection under several pathological conditions including rI/R, sepsis and mechanical ventilation [8, 23, 24]. However, our current results extend the previous findings to show that dexmedetomidine might also provide cyto-protection through modulation of F-actin and junctional complex proteins, to maintain morphology and function of pulmonary microvascular endothelial barrier (Figure 5). This is the first study to show that dexmedetomidine can provide “local direct” pulmonary protective effects at the cellular structure level besides its anti-inflammatory effect and beyond.

The permeability of PMVECs monolayer was significantly increased when challenged with different concentrations of serum (5%, 10% and 20%) from mice underwent rI/R surgery (Figure 2A), which implied that “toxicants” including pro-inflammatory mediators mentioned above and damage-associated molecular pattern molecules (DAPMs), were released from injured kidney into circulation to prompt lung damage. Arguably, this is robust evidence to indirectly re-confirm our *in vivo* findings. This injury was attenuated by dexmedetomidine in a dose-dependent manner (Figure 2B). However, dexmedetomidine at higher concentration (10 μM) lost its ability to inhibit rI/R serum induced hyper-permeability of PMVECs monolayer. The mechanisms may be involved in the activation of other unknown intracellular signals, or due to “off-target” signaling through α_1-_adrenoreceptor that remains to be clarified. Interestingly, the bidirectional or ceiling effects of dexmedetomidine on the modulation of inflammation and cyto-protection has been also demonstrated in the previous studies [25, 26].

FAK is a highly conserved cytoplasmic tyrosine kinase, involved in integrin engagement and focal adhesions assembly *via* its multiple downstream signaling pathways [27]. As a key regulator of actin cytoskeleton organization, FAK modulates cellular tension transduction, from intracellular to extracellular [19]. Furthermore, phosphorylated FAK plays a central role in cell adhesion. The present study demonstrated that dexmedetomidine produced a time- and dose-related FAK phosphorylation in PMVECs *via* α_2_-adrenoreceptor activation (Figure 3), which is consistent with previous report [16, 19]. After phosphorylation, FAK can modulate tension signaling to control actin and focal adhesion dynamics thus to promote adaptive resistance to surrounding environment deterioration [19, 28].

FAK has several tyrosine sites such as Y142 and Y925 besides Y397 [19]. The phosphorylation of different tyrosine sites may produce different physiology or pathophysiology effects. Inhibition of FAK Y397 phosphorylation abolished the protective effect of dexmedetomidine on endothelial monolayer hyper-permeability (Figure 4), indicating that dexmedetomidine acted cyto-protective effect in a FAK-activity dependent manner.

**Figure 6:**
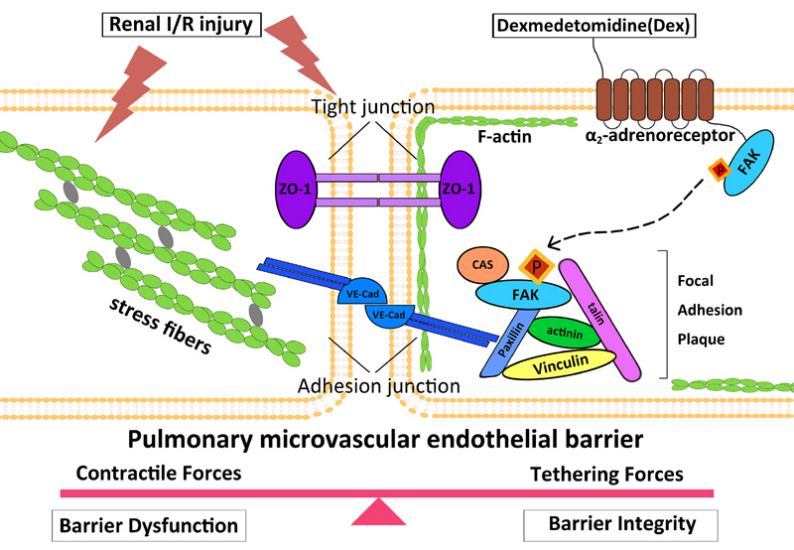
Putative mechanism of dexmedetomidine-mediated protection of pulmonary endothelial barrier involving α-adrenoreceptor/FAK pathway In this working model of endothelial barrier regulation, under baseline conditions, a balance exists between cytoskeleton contractile and cellular adhesive forces.(Left) When AKI occurs, circulating pro-inflammatory and pro-apoptosis mediators transit into pulmonary microvascular, and F-actin was organized into axially oriented stress fibers, with adherens junction (AJ) and tight junction (TJ) dissociation, followed by endothelial cells pulling apart to form paracellular gaps to culminate to barrier disruption. (Right) Dexmedetomidine binding to α_2_-adrenoreceptor activates FAK, which exhibits increased tyrosine phosphorylation at Y397 site. Phosphorylated FAK subsequently translocates to the endothelial cell periphery where they form new focal contacts associated with increased cortical actin ring, to induce junctional complex assembly to strengthen the endothelial barrier. VE-cad, vascular endothelial cadherin; ZO-1, zonaoccluden protein-1.

The endothelial barrier permeability is mainly maintained by a balance between the adhesive force produced at endothelial cell-cell junctions, and the contractile force generated at the endothelial cytoskeleton. Previously studies [18, 29] have suggested that junctional complexes, such as ZO-1 and VE-cadherin of PMVECs are important structures for the maintenance of endothelial barrier integrity through focal adhesions between endothelia cells and matrix. In many cases [30, 31], a complex interplay between cell junctional elements and focal adhesions results in rapid actin cytoskeletal remodeling. However, under pathological conditions, cytoskeletal rearrangement together with various intracellular signaling that interacts with inflammatory cells could promote release of enzymes to breakdown pulmonary microvascular endothelial barrier. This leads to intercellular gap formation to result in fluid and soluble substances moving into lung tissue from blood circulation. Fluid overload in the lung due to endothelia barrier hyper-permeability ultimately leads to pulmonary edema during acute kidney injury [32, 33]. Therefore, protecting pulmonary microvascular endothelium integrity may be a primary therapeutic strategy for the critical ill patients with harmful organ crosstalk exemplified by the current case of kidney-lung [34, 35]. When AKI occurs as shown in the current study, circulating cytokines accumulate in the pulmonary microvasculature to cause cellular F-actin rearrangement and junctional complex dissociation, and hence increase paracellular permeability. When dexmedetomidine binds to the α_2_-adrenoreceptor, then activates FAK with the increased cortical actin ring and induces junctional complex assembly that serve to further strengthen the endothelial barrier (Figure 6). Collectively, our data point to distinct roles for α_2_-adrenoreceptor/FAK activity in the control of F-actin and junctional complex, both of which are important in the regulation of vascular permeability. Interestingly, a previous clinical study demonstrated that dexmedetomidine protects lungs through anti-oxidative stress mechanisms [36], indicating that the pulmonary protection afforded by dexmedetomidine reported in the current study is very likely due to multiple cellular mechanisms.

Our work is not without limitations. Firstly, circulatory leukocyte adhesion and trafficking play an important role in acute lung injury induced by rI/R [37]. However, their role on pulmonary microvascular permeability has not been explored in the present study and warrants further study. Secondly, toxic substances and water accumulation in the circulation consequent of AKI undoubtedly contribute to other organ injury including lung injury. Dexmedetomidine can protect kidney injury in such type [8, 12] which may also partially indirectly attribute its lung protection but yet to be assessed in future study. Thirdly, the plasma concentration of dexmedetomidine has not been measured in our study. By estimation, its concentration in our experimental setting is likely much higher than the clinical targeted concentration in the plasma [38]. Therefore, it is highly plausible that such a high dose could produce pulmonary vascular constriction which may contribute to reduction in microvascular hyper-permeability. However, one can argue in general that an anesthetic drug dose administered to animals could be up to > ten times higher than given to human [39]. Finally, due to Ethics consideration, isoflurane was used for basal anesthesia and it has organoprotective effects reported elsewhere. Therefore, the pulmonary protective effect of dexmedetomidine found in this study could be distorted by the superposed effect of isoflurane. However, because of each group of animal including the controls all being give this basal anesthesia, hence this became not an issue regarding the conclusions of the current work. Nevertheless, our study showed that serum from kidney I/R mice containing enough injurious mediators including cytokines and other mediators are sufficient to damage lung cells, indicating that preventing primary organ injury is far more important.

In summary, dexmedetomidine attenuates pulmonary microvascular hyper-permeability secondary to rI/R through up-regulation of phosphorylated FAK modulated by α_2_-adrenoreceptor activation, which are likely associated with F-actin modulated endothelial intercellular junction complex to facilitate recovery following barrier disruption from injurious mediators, e.g. cytokines and DAPMS releasing from injured kidney.

## MATERIALS AND METHODS

### *In vivo* experiments

#### Animal model

The current study was approved by the Animal Care Committee of Third Military Medical University, Chong Qing, China, and was performed in accordance with the ARRIVE guideline. Male 8- to 10-week-old C57BL/6J mice weighing 20 ± 2g were purchased from the laboratory animal center of Third Military Medical University. Mice were housed in a pathogen-free condition at 25 ± 2°C, 55 ± 5% humidity, and a 12 hours light-dark cycle, with *ad libitum* access to standard pellet food and sterile water for at least 7 days before experiments. Food was removed 8 hours before the operation but free access to water was allowed.

All mice undergoing surgery were anesthetized with 1.5% isoflurane *via* mask and their body temperature was maintained at 36 ± 0.1°C with a heating pad. Renal I/R injury was induced by bilateral renal pedicle clamping for 45 minutes and reperfusion for 24 hours to produce moderate renal injury as we reported previously [12]. All animals received 0.5 ml saline intraperitoneal injection for volume supplementary every 6 hours after surgery [12] and were sacrificed 24 hours after rI/R. 1ml blood sample from abdominal aorta was obtained for each animal with or without rI/R injury. The blood was immediately placed on ice and then centrifuged (3,000g, 4°C for 15minutes) to remove cellular elements. Serum was stored in aliquots at −80°C.

#### Groups and treatments

Animals were randomly allocated into 8 groups (*n* = 5 per group):Control group (Control): no treatment, no surgery; Sham group (Sham): laparotomy was performed without renal vessels occlusion; Dexmedetomidine group (Dex): dexmedetomidine (Precedex^®^ 100μg/ml dexmedetomidine, Orion Pharma, Espoo, Finland) at 25 μg/kg was administered (intraperitoneal) without surgery; rI/Rsurgery group (rI/R surgery): laparotomy was performed and bilateral renal pedicle clamping lasted for 45 minutes, followed by reperfusion for 24 hours; Dexmedetomidine pretreated group (pre-Dex): dexmedetomidineat 25 μg/kg (intraperitoneal) was administered 15 minutes before rI/R surgery as stated above; Dexmedetomidine post-treated group (post-Dex): dexmedetomidine at 25 μg/kg was administered immediately upon release of bilateral renal pedicle clamping; Combination of atipamezole and dexmedetomidine group (Atip-Dex): atipamezole(α_2_-adrenergic antagonist, Sigma-Aldrich, St. Louis, MO, USA) at 250 μg /kg was administered 10 minutes prior to dexmedetomidine pre-treatment [12] followed by rI/R surgery; Combination of FAK inhibitor and dexmedetomidine group (FAK inhibitor-Dex): FAK inhibitor 14 (Santa Cruz, CA, USA), an inhibitor FAK phosphorylation, was administered at 30 mg/kg 5 minutes prior to dexmedetomidine pre-treatment [40] followed by renal ischemia surgery. The dose of dexmedetomidine, atipamezoleand FAK inhibitor 14 referred to the previous established protocols [12, 40].

#### Histopathology assessment

Lung tissues were harvested 24 hours after rI/R and embedded in paraffin and sectioned into 5μm sections. Sections were stained with hematoxylin/eosin and examined under a light microscope. Severity of lung injury was evaluated semi-quantitatively using a scale system [41] from 0 to 3 grades: Grade 0, normal pulmonary histology; Grade 1, mild moderate interstitial congestion and neutrophil leukocyte infiltrations; Grade 2, moderate neutrophil leukocyte infiltration, perivascular edema formation, and partial destruction of pulmonary architecture; Grade 3, complete destruction of the pulmonary architecture with dense neutrophil leukocyte infiltrations and abscess formation.

#### Pulmonary microvascular studies

Pulmonary vascular permeability was quantitatively measured by extravasation of FITC-Albumin (Sigma-Aldrich, St. Louis, MO, USA) into lung parenchyma [42]. Briefly, after 22 hours of rI/R, FITC-Albumin 5mg/kg was injected *via* tail vein of mice under surgical anesthesia. After 2 hours injection, all mice received thoracotomy under terminal anesthesia with 1.5% isoflurane *via* mask, and their lungs were harvested after 10 ml PBS transfusion to pulmonary artery for washing intravascular blood. The isolated lungs were frozen in liquid nitrogen, then homogenized and centrifuged (3,000g, 4°C for 10 minutes). Supernatant (100μl) was collected and FITC-Albumin optical density, which indirectly reflects the permeability of pulmonary microvascular, was quantitated by Varioskan Flash multimode reader (Thermo Fisher Scientific, Waltham, MA, USA) at an excitation and emission of 494 nm and 520 nm, respectively. The assessments were made by an investigator blinded to the experimental protocols.

### *In vitro* experiments

#### Cell culture

C57BL/6J mice pulmonary microvascular endothelial cells (PMVECs) were obtained from the Cell Biologics Inc. (Chicago, IL, USA). The cells were cultured at 37°C and 5% CO_2_ on gelatin-coated flasks in M-1168 complete medium as recommended. All experiments were performed on early passaged cells (passage3-6) to preserve true endothelial cell phenotype.

#### Effects of rI/R serum on PMVECs monolayers permeability

PMVECs (1 × 10^5^ cells) were seeded onto polyester membranes in the upper chamber of transwell (pore size 0.4 μm, diameter 6.5 mm, Costar, N.Y. USA) and cultured with 0.5ml of M-1168 media. Sufficient media was added to each lower chamber to cover the membrane. When cells were propagated to 100% confluence, the monolayers were exposed to fresh media without phenol red dye and FBS for 1 hour, then treated with 20% mice normal serum or different concentrations of serum (5%, 10%, 20%) from animals underwent rI/R surgery for 24 hours as described above. FITC-Albumin (10mg/ml) was added to the upper chamber of each transwell for up to 24 hours and 100 μl media sample was then collected from the lower chamber and their fluorescence were measured at 3, 6, 9, 12, 15, 18, 21 and 24 hours respectively. Sample readings were converted with a standard curve representing the FITC-Albumin concentration. The permeability coefficient (Pc) of FITC-Albumin was determined as previously described [43] as Pc = [A]/t × 1/A × V/ [L], where brackets denote FITC-albumin concentration in the lower chamber [A] or the top chamber [L], t is time (s), A is the area of the membrane (cm^2^), and V is the volume of the lower chamber.

#### Effects of dexmedetomidine on the permeability of PMVECs monolayer treated with rI/R serum

The optimal concentration (20%) of rI/R serum obtained from pilot study as above was used in the subsequent experiments. Each of the endothelial monolayers in transwell chamber was incubated with the fresh culture medium containing dexmedetomidine (0.001μM ~ 10μM) for 6 hours, before 24 hours treatment with 20% rI/R serum together with FITC-Albumin (10mg/ml). 100μl sample culture medium was collected from the lower chamber and FITC-Albumin was calculated by Varioskan Flash multimode reader. The results were expressed as percentage of Pc.

#### Effects of dexmedetomidine on FAK activity of PMVECs

PMVECs grown on gelatin-coated slips were incubated with 0.1μM dexmedetomidine or 20% rI/R serum for 1,3, 5 and 10minutes. PMVECs were fixed in 4% paraformal dehyde for 10 minutes, permeabilized with 0.2% Triton X-100 in PBS for 5 minutes, and blocked with 2% BSA in PBS for 1 hour. The changes in FAK phosphorylation were probed with phospho-tyrosine^397^FAK (P-Tyr^397^FAK) antibody (Abcam, Cambridge, MA, USA), and images were captured with a Nikon EclipseTE300 inverted microscope (Nikon, Tokyo, Japan). Fluorescence intensity of P-Tyr^397^FAK was measured with the Image J software (National Institute of Mental Health, Bethesda, Maryland, USA).

The PMVECs were starved from FBS for 1 hour then incubated with medium containing different concentrations of dexmedetomidine(0.001μM ~ 10μM) for 5minutes. This duration of incubation was determined from preliminary cell immunofluorescence experiment as above. It corresponded to the point for which the amount of P-Tyr^397^FAK reached its peak at 5 minutes. Proteins were separated by electrophoresis on 10% PAGE gels and 5% gradient gels, transferred to PVDF membrane (Millipore, Billerica, MA, USA). Membranes were blocked for 1 hour with 10% non fat milk in PBS containing 0.1% Tween 20. P-Tyr^397^FAK and FAK Primary antibodies (Abcam, Cambridge, MA, USA) were used at a concentration of 1:1,000, and HRP-coupled secondary antibodies were used at 1:5,000. Immunoblots were developed using standard ECL (Millipore, Billerica, MA, USA). Data were normalized to standardized to β-actin expression, normalized to total FAK expression, and expressed as % of control.

#### Effects of FAK activity on the rI/R serum induced PMVECs permeability

The endothelial monolayers in transwell chambers were continuously incubated with 10μM FAK inhibitor 14 for 3hours [40] prior to 0.1μM dexmedetomidine treatment for 20 minutes, followed by 20% rI/R serum challenge for 60minutes. The control cultures received neither treatments. FITC-Albumin (100mg/ml) was added in upper chamber at beginning of the treatment and 100μl sample was collected from the lower chamber to measure the real-time changes of permeability across the endothelial cell monolayer. The results were expressed as FITC-Albuminfluorescence intensity.

#### CellularF-actin, ZO-1 and VE-cadherin expression

PMVECs incubated with 10μM FAK inhibitor 14 for 3 hours before 0.1μM dexmedetomidine treatment for 20 minutes, followed by 20% rI/R serum for 60 minutes. F-actin was visualized by staining cells with FITC-conjugated phalloidin (Sigma, St. Louis, MO, USA) for 1 hour at room temperature. Proteins were extracted with cell lysis buffer, and analyzed by SDS-PAGE and immunoblots for ZO-1 and VE-cadherin expressions.

### Data analysis

Data were expressed as mean ± SD. One-way analysis of variance followed by post hoc Newman-Keuls test were used for comparison, otherwise, unpaired two-tailed Student test were used wherever appropriate. A pvalue less than 0.05 was considered to be a statistical significance.
